# Models and methods for determining the optimal number of beds in hospitals and regions: a systematic scoping review

**DOI:** 10.1186/s12913-020-5023-z

**Published:** 2020-03-06

**Authors:** Hamid Ravaghi, Saeide Alidoost, Russell Mannion, Victoria D. Bélorgeot

**Affiliations:** 1 School of Health Management & Information Sciences, Iran University of Medical Sciences, Tehran, Iran; 2 Health Services Management Centre, University of Birmingham, Birmingham, UK; 3 Public health consultant, World Health Organization, Regional Office for the Eastern Mediterranean, Cairo, Egypt

**Keywords:** Hospital capacity, Hospital beds, Method, Model, Systematic review

## Abstract

**Background:**

Determining the optimal number of hospital beds is a complex and challenging endeavor and requires models and techniques which are sensitive to the multi-level, uncertain, and dynamic variables involved. This study identifies and characterizes extant models and methods that can be used to determine the required number of beds at hospital and regional levels, comparing their advantages and challenges.

**Methods:**

A systematic search was conducted using Web of Science, Scopus, Embase and PubMed databases, with the search terms hospital bed capacity, hospital bed need, hospital, bed size, model, and method.

**Results:**

Twenty-three studies met the criteria to be included in the review. Of these studies, a total of 11 models and 5 methods were identified, mainly designed to determine hospital bed capacity at the regional level. Common determinants of the required number of hospital beds in these models included demographic changes, average length of stay, admission rates, and bed occupancy rates.

**Conclusions:**

There are no specific norms for the required number of beds at hospital and regional levels, but some of the identified models and methods may be used to estimate this number in different contexts. Moreover, it is important to consider alternative approaches to planning hospital capacity like care pathways to fix the limitations of “bed numbers”.

## Background

Hospital capacity planning is an important issue in many developed and developing countries due to the increasing costs of inpatient care, constrained resources, and the growing demand for hospital care. In many countries, bed capacity and associated indices such as bed occupancy and the ratio of beds to population are the key metrics used to determine the availability of inpatient care. The number of beds in a hospital can be conceived as a capital stock, which is influenced by the performance of medical staff and equipment [1–3]. Indeed, a shortage of available beds can seriously impact on how a hospital functions. Indeed, it is the primary cause of admission and surgery cancellations, delays in emergency admissions, early patient transfers from intensive care units, delays in patient transfers between units, and early patient discharge [2, 4]. Conversely, excess bed capacity may lead to additional costs and stagnant capital. Therefore, hospital capacity planning should consider hospital bed availability alongside issues related to productivity, and clinical efficiency [5–7].

Decisions about hospital bed capacity are made by policymakers and managers at different levels of the health system. Here, two main points should be considered: the time horizon for the number of hospital beds (short, medium, or long term) and levels of hospital bed capacity planning (strategic, tactical and operational levels). Strategic planning of hospital capacity at the regional level is concerned with anticipating long-term regional needs, while tactical planning occurs at the hospital level. The operational level focuses on determining the optimal number of beds to allocate to each hospital unit [8]. Changing hospital bed capacities is a highly sensitive political issue, involving complex negotiations, often with little use of economic indicators to inform decision making [9].

The number of beds required to provide quality healthcare depends on a range of factors including models of care, patient need, national policies, and local circumstances. National policies focus on how to meet this demand. Local circumstances include internal hospital processes, the availability of other services, and influence demand management at the local level. Given that the interaction of these factors varies over time and within countries, the number of beds required to deliver health services also varies [10].

Hospital bed capacity cannot be determined without taking into account hospital needs, policies, existing services, staffing patterns, and other relevant aspects. Any projections regarding hospital services and future inpatient care should also be alert to wider health system changes (e.g. demographic trends, the substitution of day care for inpatient care, and changes in average length of stay) [11]. This is because in many healthcare systems there is concerted effort to shift towards more ambulatory forms of care [12, 13]. New technologies make it possible for more complex interventions to be undertaken in ambulatory or outpatient settings. Therefore, estimating the required number of hospital beds is a challenging task as many complex factors need to be taken into account, some of which are difficult to model and predict in advance. This creates the need for sophisticated modelling and sensitivity analyses to better estimate the numbers of hospital beds required to meet changing patient needs [14]. Because of these changes and complexities, we need modular architecture and to be flexible in the design of hospitals. Therefore, heavily top-down approaches are inappropriate and it is important to allow some level of local adaptation in the designing of hospitals. Most studies focus on the optimal allocation of hospital beds and use tools such as simulation methods for this purpose. The results of these studies show that this model can be a helpful tool for hospital managers in the planning and management of hospital beds [14–16].

Against this background, the purpose of this article is to review critically the various models and methods that have been developed and deployed for determining the required number of hospital beds at hospital and regional levels, as part of strategic and tactical planning. In this review, identified models and methods will be described and compared based on their key affecting factors, their advantages, and their challenges. This review follows the methodology of a scoping review, as the aim of the present research is to provide an overview of models and methods, rather than to answer specific questions.

## Methods

The methodology used follows the framework for scoping reviews developed by Arksey and O’Malley (2005). Arksey and O’Malley’s (2005) framework distinguishes between four types of scoping reviews. The first considers the extent, range, and nature of the available literature. The second aims to determine the value of conducting a full systematic review. The third summarizes and disseminates the findings of the existing literature. The fourth is designed to identify research gaps. The present review, conducted between January and September, 2018, research followed the methodology for the third type through a systematic search, summarizing and disseminating research findings related to the determination of the required number of hospital beds at hospital and regional levels [17]. One of the main differences between scoping reviews and systematic reviews is that, based on Arksey and O’Malley’s framework (2005), quality appraisal is not required in scoping reviews. Therefore, the quality of the studies included in this review was not assessed.

As indicated in the framework by Arksey and O’Malley (2005), we first identified the research questions: What models and methods are applied to determine the optimal number of hospital beds? What are the characteristics of these models and methods? What are the advantages and challenges of these models and methods? Second, we conducted a systematic search and identified studies relevant to the research questions. After electronic and hand searching, identified studies were screened based on the inclusion criteria, and eligible studies were selected. The content of the included studies was then coded and their data was extracted based on the research questions. Finally, the reviewers analyzed the findings, and summarized and reported the results.

### Eligibility criteria

Studies providing a method or model for determining the required number of hospital beds at hospital or regional levels (i.e. strategic or tactical levels of hospital capacity planning) were included. Studies on nursing, residential, or long-term care facilities were excluded. Studies estimating the number of hospital beds within hospitals to discuss their optimal allocation to departments (i.e. operational level of hospital capacity planning) were also excluded. Therefore, the research focuses on strategic and tactical levels of hospital capacity planning rather than on the operational level. The search identified studies published in any language between January 1st, 2000, and January 31st, 2018.

### Information sources

A range of sources were used to identify the relevant studies for review: electronic database searches, contacting experts and authors and a thorough check of the references of the included articles. Four electronic databases were searched systematically: Web of Science, Scopus, Embase, and PubMed. Hospital bed capacity, hospital bed need, hospital, bed size, model, and method, were used as free text terms, MeSH terms, and Emtree subject headings to search each database through titles, abstracts, and keywords. In addition, the authors searched Google and Google Scholar using the same terms, scanned references of relevant studies, and contacted experts to identify additional research and grey literature.

### Search

The search strategy used to search the PubMed database is provided in Table 1.

**Table 1:** The search strategy for PubMed

Database	Search strategy	Results
PubMed	((((hospital [Title/Abstract] AND bed [Title/Abstract] AND capacit*[Title/Abstract]))) OR (hospital [Title/Abstract] AND bed [Title/Abstract] AND number [Title/Abstract]))) OR (hospital [Title/Abstract] AND bed [Title/Abstract] AND size [Title/Abstract]))))) AND (model [Title/Abstract] OR method [Title/Abstract])	756

### Selection of sources of evidence

Following the systematic search, titles and abstracts of identified studies were independently examined by two of the authors, and then full text articles were reviewed to determine eligibility. Any disagreement between reviewers on whether to include or exclude a study were discussed until they came to a consensus.

### Data charting process

The content of each study was coded and the characteristics, advantages and challenges of models and methods were extracted. Data extraction was conducted independently by two reviewers and any disagreements were resolved through discussion. A translator was available on the team in case a study in a language other than English had been included.

### Data items

The data extraction form contained the following items: author, year, country, study design, proposed method(s) or model(s), the characteristics of models or methods, key affecting factors, advantages and challenges.

### Synthesis of results

Data extracted from included studies were synthesized through descriptive and narrative synthesis. Content analysis was used to explore key affecting factors, advantages and challenges for each model and method.

## Results

### Selection of sources of evidence

**Fig. 1:**
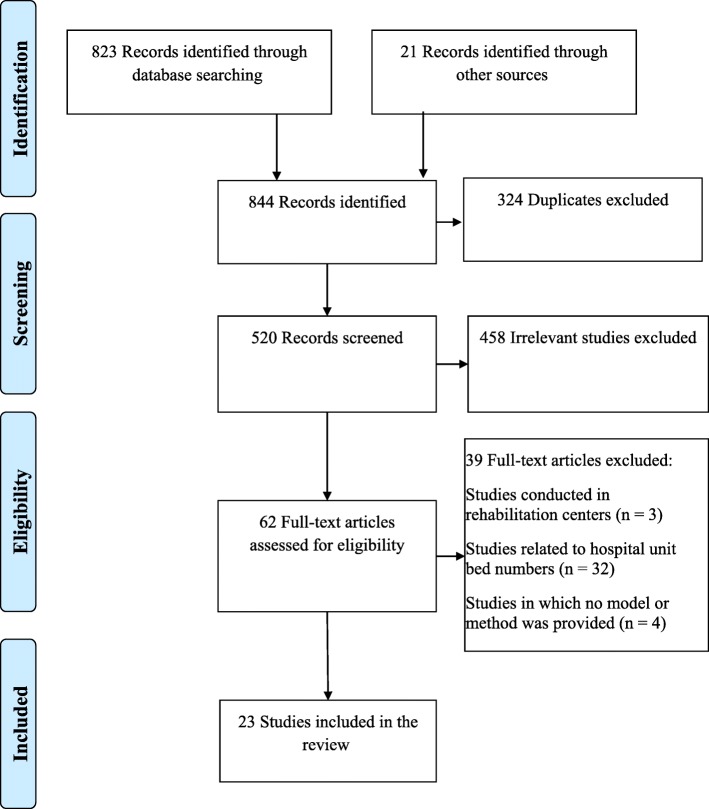
PRISMA flow diagram representing the study selection process. From: Models and Methods for Determining the Optimal Number of Beds in Hospitals and Regions: A Systematic Scoping Review

In total, 823 studies were identified through the systematic database search, and 21 via other sources: 10 studies from the grey literature, 8 studies from reference lists, and 3 studies following the advice of experts and authors. After removing duplicates, the titles and abstracts of 520 studies were examined in detail.458 of these studies were excluded due to failing to meet the inclusion criteria. The remaining 62 studies were subsequently assessed, and their reference lists were thoroughly investigated. 23 studies were ultimately included in the review. The study selection process is detailed schematically in Fig. 1.

### Characteristics of sources of evidence

Data extraction revealed that the majority of the included studies had been conducted after 2000, more specifically between 2000 and 2005 [4, 5, 7, 18–26]. Most of the studies concerned high-income countries, in particular France [5–7, 21]. The language of the included studies was English, with the exception of the studies by Oettli and Weaver which was published in French [19, 27]. A summary of the characteristics of the included studies is presented in Table 2.

**Table 2:** General characteristics of included studies

Characteristics		Number	Percent (%)
Publication year	Before 2000 [28]	1	4.3
	2000–2005 [4, 5, 7, 18–26]	12	52
	2006–2010 [6, 27, 29–31]	5	21.8
	2011–2015 [32–34]	3	13
	2016-present [10, 16]	2	8.7
Publication type	Journal article [4–7, 16, 18, 21–23, 25, 26, 28, 30–32]	15	65.2
	Report [10, 19, 20, 24, 27]	5	21.8
	Conference proceeding [29, 33]	2	8.6
	Thesis [34]	1	4.3
Study setting	France [5–7, 21]	4	17.5
	Switzerland [19, 27, 32]	3	13
	Canada [18, 24, 30]	3	13
	Iran [16, 20, 26]	3	13
	United States [4, 31]	2	8.7
	United Kingdom [10, 28]	2	8.7
	Israel [34], Scotland [25], New Zealand [22], Brazil [33], Greece [23], Singapore [29]	6	26.1

### Synthesis of results

A total of 11 models and 5 methods were identified in the included studies. The models and methods were designed to calculate the total number of hospital beds required at hospital and regional levels. Models and methods are tools used to solve problems in socio-technical systems such as hospitals. More particularly, a model is a comprehensive and systematic approach representing the present or future state of a system in a simple way. While a model may include theoretical principles and procedural sequences or techniques, a method is a process by which a calculation or estimation is made. In this research for example, methods do not include theoretical factors affecting the required number of hospital beds. Therefore, factors such as demographic changes, technological advances, and models of care, are not taking into account in methods [35]. General characteristics of identified models and methods, and key affecting factors considered by the models and methods are provided in Tables 3 and 4.

**Table 3:** Key affecting factors considered by the models and methods identified

Identified models and methods		F^1^	ALOS^2^	BOR^3^	CBN^4^	AR^5^	P^6^	ORU^7^	RPR^8^	WT^9^	HR^10^	PT^11^	D.P^12^	T.A^13^	F.L^14^	Countries using these methods/models
Models	Michigan’s Bed Need methodology	1	*	*		*	*									United States
	The Status Quo model	2					*									Canada
	The Current Use Projection model	3		*			*		*							Canada
	The Trends in Acute Care Bed Use model	3	*	*		*	*	*	*							Canada
	The Israeli model	1		*		*	*		*							Israel
	The Greater Glasgow model	1		*			*			*		*				Scotland
	The Swiss Health Observatory (SHO) model	3	*	*		*	*	*								Switzerland
	Lausanne University Hospital (CHUV) model	1	*	*		*	*									Switzerland
	Basic scenario model	1	*	*		*	*									Switzerland
	Capacity model	1	*				*	*	*			*	*	*	*	New Zealand
	Score model	3		*		*						*				France
Methods	Formula method	8	*	*			*				*					Brazil, Canada, France, Greece, Iran, Switzerland, United States, United Kingdom
	Ratio method	3	*			*										France, Iran, United Kingdom
	Regression method	2	*	*		*										Canada, Singapore
	Method using the distribution of present patients	1		*												France
	Simulation method	3	*	*	*	*										Iran, Canada

^1^ Frequency of use

^2^ Average length of stay

^3^ Bed occupancy rate

^4^ Current bed numbers

^5^ Admission rates

^6^ Population

^7^ Out-of-region use (inter-regional flows)

^8^ Region of patient residence (sub-regional access)

^9^ Waiting time

^10^ Hospitalization rates

^11^ Patient transfer to other providers

^12^ Disease prevalence

^13^ Technology advances

^14^ Funding level

**Table 4:** General characteristics of identified models and methods used to determine required numbers of hospital beds

Model/ method (Reference)	Country	Description
Michigan’s Bed Need model [31]	United States	• Adopted in 1997 by the State-Wide Health Planning Commission • Based on the examination of demographic changes by age group and age-specific rates of hospital care use • Use of the ratio-based method • Suitable for areas and regions (sub-areas and sub-regions)
The Status Quo model [18, 34]	Canada	• Presented in a study by The Manitoba Centre for Health Policy (MCHP) • Based on changes in population size • Considers that per capita utilization of hospital services is constant • Considers that changes in hospital bed utilization rates are equal to changes in population size (e.g. a 4% increase in population size should increase bed numbers by 4%)
Current Use Projection Model [18, 24, 34]	Canada	• Presented in a study by MCHP • Based on demographic changes (population size, age and sex composition, and region of residence), and on current hospital bed utilization rates (based on three years of data) • Use of the ratio-based method
The Trends in Acute Care Bed Use model [18, 24, 34]	Canada	• Presented in a study by MCHP • For the next 10 years, based on demographic changes (population size, age and sex composition, and region of residence) and trends in utilization of hospital services • The revised version of this model cannot project beyond 3 years • Considers that average length of stay and inpatient admission rates are decreasing • Use of Poisson regression
Israeli model [34]	Israel	• Similar to the Trends in Acute Care Bed Use model • Based on demographic changes (population size and growth, age and sex composition, and region of residence) and current patterns of hospital service utilization
The Greater Glasgow model [25, 34]	Scotland	• Combines top-down and bottom-up approaches • Bottom-up approach: identification of 14 clinical groups by examining care pathways and models of care • Top-down approach: Study of the following eight criteria: performance improvement (hospital goal to become a “top” hospital), bed occupancy rates by specialty, demographic changes (particularly age distribution), shift to community facilities (e.g. for patients with long lengths of stay), waiting times, emergency care (and new methods for emergency patients), increase in number of emergency patients, and geographic flows (patterns of patient flow between hospitals in different regions)
The Swiss Health Observatory (SHO) model [19, 27, 32]	Switzerland	• Presented in 2000 and revised in 2009 • Consists of two stages: development of scenarios by area (canton), and estimation of future needs of hospital care based on Diagnosis-Related Groups (DRG) • Development of different scenarios based on key uncertainties (admission rates, average length of stay, demographic changes) • Considers that average lengths of stay will decrease in the next 10 years • Use of the ratio-based method • Suitable for determining bed requirements at the regional level
Lausanne University Hospital (CHUV) model [32]	Switzerland	• Modeled after the Swiss Health Observatory (SHO) model • Based on scenarios and key uncertainties (admission rates, average length of stay, demographic changes) • Use of the ratio-based method • Suitable for determining bed requirements at the hospital level
Basic scenario model [32]	Switzerland	• Uses scenarios based on demographic changes • Use of the ratio-based method • Suitable for determining bed requirements at the regional level
Capacity model [22]	New Zealand	• Based on a mathematical iterative linear equation, the examination of current hospital bed utilization rates, and factors affecting future rates • Considers trends of demand for services, factors related to demand (population growth, disease prevalence, transfers to or from the private sector), supply-side factors (technological advances, changes in funding, length of stay and patients’ transfers), external factors like inter-regional flows and sub-regional equitable access (SREA) • Prediction of bed requirements based on the cumulative impact of the above factors on baseline bed use for each service • Use of Monte Carlo analysis
Score model [5–7]	France	• Based on a score constructed with three parameters: bed occupancy rate (measure of efficiency), number of transfers due to lack of beds (measure of clinical effectiveness), and number of days without the possibility for unscheduled admissions (measure of accessibility) • The number of beds is optimal when the mean and standard deviation of this score is the lowest • The number of beds is optimal if the following parameters have a low value: the number of days for which the number of unoccupied beds exceeds a given threshold (efficiency), the number of patients transferred due to the lack of bed availability (clinical effectiveness), and the number of days without the possibility for unscheduled admissions (availability) • Using this model increases availability and clinical effectiveness, but reduces efficiency • Application of a simulation method using software
Ratio Method [9, 21, 26, 36]	France / UK / Iran / OECD countries	• Introduced by Jung and Streeter in 1977 • Based on the ratio of the total length of stay (average length of stay × number of patients) to period duration
Formula method [4, 18, 19, 21, 23, 26, 28, 30, 33, 37]	United States / United Kingdom / France / Switzerland / Iran / Greece / Brazil / Canada	• Introduced in 1984 • Based on target occupancy rates (80–85% for large hospitals and 45% for small hospitals) • Calculated by dividing the total length of stay (average length of stay × admission rate × projected population size) by (period duration × target bed occupancy rate)
Method using the distribution of present patients [21]	France	• Based on the distribution of occupied beds. For example, the proportion of days in which 0–5 beds, 6–10 beds, 11–15 beds, etc. are occupied, and the number of beds occupied on most days, indicates the number of beds needed
Simulation method [16, 18, 26]	Iran / Canada	• Based on admission rates, discharge rates, average length of stay, and distribution of occupied beds for each day • Used alone or in combination with other methods
Regression method [18, 29]	Canada /Singapore	• Based on the number of occupied beds (dependent variable) as a function of independent variables such as occupied beds in past weeks, admission rates, length of stay, and emergency admissions

In this study, identified models and methods are compared based on their advantages and challenges. Some of the advantages include: easy estimation, highly flexible, considers hospital performance and efficiency, various scenarios, technological advances, trends in hospital service utilization, and seasonal effects. Some of the challenges investigated in the models and methods include low accuracy, overestimation of required bed numbers, and difficulty of demographic predictions. A comparative overview of the identified models and methods is presented in Tables 5 and 6.

**Table 5:** Comparison of the models’ advantages and challenges

		Score model	Capacity model	Basic scenario model	CHUV model	SHO model	The Greater Glasgow model	Israeli model	The Trends in Acute Care Bed Use model	Current Use Projection Model	The Status Quo model	Michigan’s Bed Need model
Advantages	Investigates future demographic changes			*	*	*	*	*	*	*		*
	Easy estimation	*									*	
	Considers regional population distribution							*	*	*		
	Considers population age and sex composition			*			*	*	*	*		
	Estimates bed requirements by the type of clinical specialties		*		*	*	*	*	*	*		*
	Considers trends in hospital service utilization		*		*	*			*			
	Considers performance and efficiency of hospital						*					
	Accounts for patient migration					*	*		*			
	Considers medical and technological advances						*		*			
	Considers care models and standards		*				*					
	Considers emergency cases and future trends						*					
	Considers various scenarios		*	*	*	*						
	Considers seasonal effects		*									
	Easy to use	*										
	A software is available	*										
challenges	Requires accurate and comprehensive data											*
	Difficulty of demographic predictions	NA	NA	*	*	*	*	*	*	*	NA	*
	Difficulty of predicting patterns of hospital service utilization		*		*	*			*			
	Does not account for policy changes overtimes	*	*	*	*	*	*	*	*	*	*	*
	Overestimation of required bed numbers							*	*			
	Difficulty of mapping scenarios	NA	*	*	*	*	NA	NA	NA	NA	NA	NA
	Does not assign weightings to the parameters	*	*	*	*	*	*	*	*	*	*	*
	Needs a simulation software	*										

*NA* Not Applicable

**Table 6:** Comparison of the methods’ advantages and challenges

Comparative aspects		Ratio Method	Formula method	Method using the distribution of present patients	Simulation method	Regression method
Advantages	Investigates hospital conditions				*	
	Easy estimation	*	*	*		*
	Highly flexible					*
	Accounts for changes in the average length of stay				*	
	Accounts for factors affecting the average length of stay				*	
	Estimates bed requirements by clinical specialties	*	*	*	*	*
	Requires little time	*				
challenges	Does not consider factors affecting the demand for hospital care	*	*	*	*	*
	Does not consider factors affecting the supply of hospital care	*	*	*	*	*
	Requires accurate and comprehensive data				*	*
	Time-consuming and costly				*	
	Does not account for the dynamics of certain key parameters, like demographic changes and patterns of hospital service utilization	*	*	*	*	*
	Low accuracy			*		*

Population, average length of stay, admission rates, and bed occupancy rates are common variables included in the design of models and methods. Demographic changes are included in every model, with the exception of the Capacity Model and the Score Model. The focus on the use of new medical technologies and out-of-region use of hospital services in determining hospital bed capacity is very limited in all models, although in recent years these factors appear to have received more sustained attention.

Most models use population age groups, clinical specialties (or diagnosis-related groups), and trends in use of hospital services (e.g. average length of stay, admission rates) to estimate required numbers of hospital beds. However, traditional methods such as the Ratio method and the Formula method do not consider these elements when estimating required bed numbers, and as a result, traditional methods are often combined with more recent models. The analysis of models and methods reveals that most models assume policies and technologies will not change, resulting in an overestimation of bed numbers. The Capacity Model is the only model or method considering both the supply and demand of hospital services and external factors. A description of the different models and methods are elaborated in Table 4.

## Discussion

Determining the optimal numbers of hospital beds is a complex task which is undertaken at different levels of the health system. Balancing costs, accessibility, and quality remains a key challenge. No specific methodology has been identified in our review as being the most suitable to determine the optimal number of hospital beds. As our review has shown, a wide variety of models and methods exist for this purpose, and several factors affecting required numbers of hospital beds have been identified within them. The health system has control over some of these factors, such as the effectiveness and quality of hospital services, and alternatives to hospital care for example. However, factors external to the health system, such as disease patterns and demographic changes, are harder to predict and control. External factors and factors related to supply and demand are presented in Table 7. Several dimensions should be taken into account for optimal capacity planning, including the accessibility of the required data, and the most appropriate ways of quantifying factors affecting bed requirements [4, 24, 38, 39]. And it is up to managers and policymakers to select the most appropriate approach depending on their specific goals, the desired timeline, the level of bed capacity planning (hospital or regional), the national context, and the availability of comprehensive data [8].

**Table 7:** Factors affecting the required number of hospital beds

Demand factors	Supply factors	External factors
Admission rates	Average length of stay	Political pressures
Hospitalization rates	Current bed numbers	Policy changes
Population changes	Waiting time	Sub-regional access
Seasonal effects	Bed occupancy rate	Inter-regional flows
Epidemiological changes such as diseases prevalence	Medical and technological advances	
Emergency cases and future emergency trends	Hospital efficiency	
	Clinical and service performance	
Region of patient residence (rural or urban)	Alternatives to hospital care	
	Patient transfers to other providers	
	Funding level	
	The type of care (surgical or non-surgical)	

All of the models and methods identified in the present review account for at least one of these factors. Demographics, admission rates, lengths of stay, and bed occupancy rates were most commonly identified. The ratio-based method and formula method are traditional approaches to determine bed requirements based on demand (inpatient admission rates) and supply (length of stay) of hospital care. These approaches are typically most suitable for hospital-level planning. However, they have some limitations, which reduce their applicability. For example, traditional approaches assume steady state supply and demand and do not account for a number of factors such as demographic changes and patient migration. They are therefore used in combination with other methods and models in most countries. The formula method which considers target bed occupancy rates is used as the foundation for many models.

Most models are intended for medium- or long-term estimates at the regional level. However, some models, such as the Lausanne University Hospital (CHUV) model, have been designed for the hospital level. Hospital-level models are often based on regional-level models. Hence, it is possible for hospital managers to use models intended for macro-level use, depending on the unique conditions of their hospitals [32].

In most models, existing and future hospital care use trends have been examined. These have been examined in a variety of ways depending on the time horizon of interest. In some models such as the Trends in Acute Care Bed Use model, the Lausanne University Hospital (CHUV) model, and the Capacity Model, trends in hospital care use are examined through population age and sex composition, region of residence (urban or rural), and clinical specialty (or diagnosis-related group). Trends for surgical and non-surgical services, are also considered, based on a specific time horizon. However, in some models, for example the Swiss health observatory (SHO) model and basic scenario model admission rates are assumed to be constant. This has serious limitations given the occurrence of demographic and epidemiological changes. It is important to note that the number of beds estimated by these models is greater than the number of beds estimated by the models that account for changes and trends in demand [18, 24, 34].

Overall, population is the most important factor to take into account for hospital bed capacity planning. Additionally, age and sex composition, as well as regional distribution, should be considered alongside population size to obtain an accurate representation of the population. These factors may affect the demand for hospital care (admission rates) and the supply of services (average length of stay). Patient flows should also factored in as patients may use hospital services in different regions [24]. In most models, population composition and demographic changes are taken into account, and demographic projections are made by age group, sex, and region of residence for a certain time horizon [18, 40]. However, if demographic projections are not accurate or patterns of hospital service utilization vary from predicted trends, models will not yield accurate results [24]. Furthermore, population growth and aging can affect the demand for hospital care. Older people are typically the main users of hospital care, increasing admission rates and the average length of stay. However, technological advances and e-health may reduce the average length of stay in the future [32].

Furthermore, evidence shows that the mere use of demographics can lead to overestimation or underestimation of required bed numbers. Therefore, in addition to demographic changes, the impact of technological advances, periodic crises, emerging diseases, and epidemiology must be accounted for. Existing evidence suggests that technological advances and new interventions will reduce the need for hospital beds in the future and shift care towards the ambulatory or outpatient settings. Thus, these should be considered alongside demographic and epidemiological changes in order to allow for more accurate estimations of required numbers of hospital beds. The impact of technological advances on the number of required hospital beds is difficult to quantify [4, 18, 41]. Advances in medical technology and reduced dependency on hospital care in the United Kingdom have decreased the need for inpatient care, and thus reduced the number of hospital beds. A shift in patient care from hospitals to community settings has led to the largest reduction in the number of hospital beds for mental health patients and patients with learning disabilities. The number of hospital beds in England in general and acute services has dropped by 43%, mainly due to the dramatic decrease of beds for the long-term care of older people. In addition, medical innovation and the increase in day-case surgery has had a significant impact in this regard. The number of maternity beds has dropped by about 51% as a result of changes in length of stay. However, the number of day-only beds has increased by five times due to the rise in day-case surgery [10].

Admission rates in specialties such as infectious diseases or injuries demonstrate unique patterns, and may not follow demographic changes or population age and sex composition [42]. Therefore, in addition to demographic and technological changes, climate and seasonal changes should be examined. Indeed, the relationship between health and the environment has been described, and factors such as air temperature can contribute to the development and spread of various diseases [22]. Climate and seasonal changes must also be considered to improve the management of hospital bed capacity. Therefore, demographic changes are only a part of the complex equation behind bed estimations [22, 42]. Moreover, bed requirement estimates should not depend on a single scenario. Indeed, it may be advisable to consider multiple scenarios based on predictions of one or several key factors. In the Swiss Health Observatory Model for example, nine scenarios are developed based on demographic changes, lengths of stay, and admission rates, and the required number of beds are calculated for each scenario using the formula method. This model predicts that lengths of stay will decrease in the future and examines this trend across different scenarios [19, 27, 32]. Therefore, it is necessary to assess the potential consequences of an erroneous estimation of hospital bed numbers. Average length of stay is another key factor in determining the required number of hospital beds and proxy for resource utilization and hospital bed efficiency. Length of stay is affected by a number of factors, such as patient characteristics (age, insurance type), admission status (elective or emergency, day of admission), admission season, and time for conducting consultation services and delivering laboratory services [16, 43]. Given technological advances, policies for efficiency improvement, shifts from inpatient to outpatient surgeries, and prospective payment systems, average lengths of stay are expected to decrease in the future [5, 23, 34]. Therefore, it is not reasonable to assume that this will remain constant when calculating numbers of beds required. Indeed, planning the optimal number of beds should be based on more realistic assumptions about the trends in average lengths of stay and should differentiate between distinct clinical groups (diagnosis-related groups) and patient age groups. The internal hospital environment and regional conditions are also important to consider. For example, reduction in the average length of stay in university hospitals is unlikely, due to the complexity of diseases they treat and services they deliver [32, 44].

Although most models in the review recommend that the average length of stay should be assumed to vary, bed occupancy rates are usually considered constant. Desirable bed occupancy rates vary depending on perspective and can be decreased with reductions in admission rates and average lengths of stay (Table 7). However, the trend is assumed to remain constant. Additionally, the target bed occupancy rate is usually considered to be 80–85% of the total bed capacity for large hospitals, and 45% for small hospitals (due to lack of economies of scale) [4]. It is important to note that some hospitals need overflow beds, due to the conditions and epidemiological characteristics of the region, in case of emergencies (these beds can be unstaffed at other times) [4]. Evidence shows that bed occupancy is considered to be higher in hospitals and units with overflow beds, and changes in demand and admission rates affect it more than hospital efficiency [4, 30]. Bed occupancy targets are set to control the supply of hospital beds and costs, and to identify shortages [4]. In addition to target bed occupancy rates, planning and management of bed capacity must consider standards of clinical and service performance, like average waiting times for beds, so that patients can be placed in the right beds at the right time. Thus, managers and policymakers must determine hospital capacity based on performance measures [4].

In addition to factors related to the supply and demand for services, external issues such as sub-regional equitable access, inter-regional patient flows, funding policies, staffing, and availability of standards must be taken into consideration. These factors are examined in a few models such as the capacity model [10, 22]. One of the important issues affecting on the hospital bed number in one region is decision making on expanding the existing hospitals or constructing new small hospitals. This is a political issue and also very difficult for policy makers to choose between them. In this situation, there is a challenge between efficiency and accessibility of hospital services. So, policymakers should consider all external elements, alongside demand and supply-side factors to determine required numbers of hospital beds at hospital or regional levels. It is also necessary to develop different scenarios based on these factors, and to use a combination of methods and models to estimate bed requirements [32]. However, hospital capacity planning should not be limited to these components and must account for variations in bed numbers. Evidence suggests that the supply of hospital beds increases the demand for hospital services (Roemer’s Law), and that this manifest in higher admission rates, longer stays, or a mixture of both [45, 46]. However, an increase in the number of beds in long-term care facilities reduces the average length of stay in acute care hospitals, and increases the rate of discharge from acute care hospitals to other facilities. This issue must be taken into consideration to determine the optimal number of beds in acute care hospitals and long-term care hospitals within a region [47].

In addition to supplying required numbers of hospital beds in the short and medium terms, managers and policymakers require information about the most effective strategies to reduce the need for hospital beds in the future. The most effective, way of doing this appears to be through health promotion and disease and disability prevention [38]. However, to reduce bed needs in the short term, health systems can use a range of interventions that seek to reduce inpatient admissions and facilitate inpatient discharge. Avoiding emergency admissions by establishing medical observation units, avoiding non-urgent admissions through day care, and shifting inpatient care to ambulatory care are the most effective strategies for reducing inpatient admissions [10, 11, 24, 26, 48]. Discharge planning from hospital to home is another effective strategy in reducing the need for hospital beds, as it decreases lengths of stay and the level of unplanned readmissions, by facilitating patient discharge and shifting care from hospitals to homes [49]. Managers and policymakers should therefore be concerned with ensuring appropriate bed use for different specialties before seeking to increase the number of hospital beds [50]. Furthermore, integrated care is another strategy to strengthen health systems and reduce the need for inpatient care by focusing on individual needs, collaboration, and coordination between specialties and among health care providers [51]. There are three levels of integration that have been shown to be effective in reducing the need for hospital beds: the macro-level (provision of integrated care to an entire population), the meso-level (provision of integrated care to a particular care group or population with the same disease or condition), and the micro-level (coordination of care for individual carers and patients). Integrated care pathways are tools developed by multidisciplinary teams for pre- and post-admission care. A number of studies have shown that these pathways can reduce non-urgent admissions and lengths of stay and, consequently, reduce the need for hospital beds [52–54].

### Limitation

Besides hospital bed capacity planning at the regional and hospital levels, hospital bed capacity by different inpatient units must be determined to allow for optimal allocation. However, in our review, the latter (operational level of capacity planning) was not examined because of time constraints. This calls for a study of methods and models related to the operational level of hospital capacity planning and optimal bed distribution among different hospital units.

## Conclusions

Decisions about the optimal number of hospital beds can be made at hospital or regional levels. However, given the interaction between variables influencing bed number requirements, it is recommended to determine the required number of hospital beds by accounting for demographic factors, clinical specialty groups (surgical and non-surgical), inter-regional access to services, efficiency, and standards for provision of care. Currently, there are no specific standards for the required number of beds at hospital and regional levels. Policymakers can use various existing models and methods to estimate the optimal number of beds on the basis of country conditions, demographic and epidemiological changes, geographical characteristics, political and socio-economic factors, and by accessing accurate and comprehensive data. Therefore, strategic planning is necessary for managers and policymakers to gain a clearer understanding of the future of hospitals and of their optimal number of beds. In addition to supplying bed requirements in the medium term, policies should also be implemented that are designed to reduce the future need for hospital beds, especially through the expansion of primary health care and health promotion activities.

## Data Availability

Not applicable.

## Abbreviations

CHUV: Centre Hospitalier Universitaire Vaudois (The Lausanne University Hospital)
MCHP: The Manitoba Centre for Health Policy
SHO: The Swiss Health Observatory
SREA: Sub-Regional Equitable Access
